# Topsoil and Deep Soil Organic Carbon Concentration and Stability Vary with Aggregate Size and Vegetation Type in Subtropical China

**DOI:** 10.1371/journal.pone.0139380

**Published:** 2015-09-29

**Authors:** Xiang-Min Fang, Fu-Sheng Chen, Song-Ze Wan, Qing-Pei Yang, Jian-Min Shi

**Affiliations:** Collaborative Innovation Center of Jiangxi Typical Trees Cultivation and Utilization, College of Forestry, Jiangxi Agricultural University, Nanchang, 330045, China; Shandong University, CHINA

## Abstract

The impact of reforestation on soil organic carbon (OC), especially in deep layer, is poorly understood and deep soil OC stabilization in relation with aggregation and vegetation type in afforested area is unknown. Here, we collected topsoil (0–15 cm) and deep soil (30–45 cm) from six paired coniferous forests (CF) and broad-leaved forests (BF) reforested in the early 1990s in subtropical China. Soil aggregates were separated by size by dry sieving and OC stability was measured by closed-jar alkali-absorption in 71 incubation days. Soil OC concentration and mean weight diameter were higher in BF than CF. The cumulative carbon mineralization (C_min_, mg CO_2_-C kg^-1^ soil) varied with aggregate size in BF and CF topsoils, and in deep soil, it was higher in larger aggregates than in smaller aggregates in BF, but not CF. The percentage of soil OC mineralized (SOC_min_, % SOC) was in general higher in larger aggregates than in smaller aggregates. Meanwhile, SOC_min_ was greater in CF than in BF at topsoil and deep soil aggregates. In comparison to topsoil, deep soil aggregates generally exhibited a lower C_min_, and higher SOC_min_. Total nitrogen (N) and the ratio of carbon to phosphorus (C/P) were generally higher in BF than in CF in topsoil and deep soil aggregates, while the same trend of N/P was only found in deep soil aggregates. Moreover, the SOC_min_ negatively correlated with OC, total N, C/P and N/P. This work suggests that reforested vegetation type might play an important role in soil OC storage through internal nutrient cycling. Soil depth and aggregate size influenced OC stability, and deep soil OC stability could be altered by vegetation reforested about 20 years.

## Introduction

Forest stores more than 80% of aboveground and up to 70% of belowground terrestrial carbon [1]. Reforestation or afforestation has the potential to contribute to C storage directly through living biomass and soil organic carbon (OC) accumulation [2,3] and indirectly as an alternative to fossil fuels for energy generation/production [4,5]. Silver et al. [6] reported that the aboveground biomass and soil OC increased the first 100 years after reforestation and soil OC accumulated at faster rates during the first 20 years (1.3 Mg C ha^-1^ yr^-1^) and at a rate of 0.41 Mg ha^-1^ yr^-1^ over a 100-year period in tropical area. Moreover, soil OC is an indicator of soil productivity and ecosystem stability [7,8], which can be used to evaluate the effectiveness of reforestation. In order to maximize the potential for carbon sequestration, the factors influencing soil OC in plantation require further analysis. Assessing the contribution of reforestation to soil OC is complicated by the influence of additional factors such as tree species, climate conditions, soil properties and exogenous disturbances [4,5,9]. Although the impact of reforestation on soil OC has previously been investigated *in situ* and in laboratories [10], the mechanisms by how soil OC is influenced by reforestation is not fully understood [11].

Forest soil carbon stock is often measured to a depth of 1 m. However, most studies on soil OC accumulation and stabilization mechanisms mainly focus on the topsoil (0–5 cm or 0–10 cm), which is richer in OC, and easier to sample [12,13,14]. Recently deeper stocks of OC have been the focus of increasing interest, as almost half of the total OC within the first meter of soil was found at depths below 30 cm [13,15]. Even though there is no standard definition of topsoil and deep soil in the related literatures, operationally it refers to the depth of soil rather thanthe formation of pedogenic horizons and 0–20 cm soil layer is often called topsoil and below 30 cm is deep soil [10,13,16]. It has been found that the conditions of carbon stabilization between deep soil and topsoil may be discrepant [17,18] and deep soil OC is characterized by mean residence time up to several thousand years [19]. With rising atmospheric CO_2_ posing a threat to the global climate, it is important to understand the mechanisms of OC storage and stabilization in deep soils, especially in subtropical and tropical forests [20].

The turnover rate of soil OC was found to vary with soil particle-size [21,22,23]. Atmospheric CO_2_ may be sequestered in deep soil by the formation of silt- and clay-associated soil OC [22]. Organic matters within soil aggregates generally decompose less rapidly than those outside of aggregates [24], which indicates physical protection is also a key factor contributing to stabilization of OC in deep soils [7]. Drury et al. [25] found that CO_2_ production decreased with increasing aggregate size in laboratory incubation and the size distribution of soil aggregate had substantial impacts on the CO_2_ production. In addition, interaction between mineral phase and chemical composition was reported to be the main stabilization mechanism in acid soils [26], however the precise role of aggregation in long-term stabilization of organic matter in deep soils remains to be determined.

Globally, roughly 140 million hectares of land had been reforested before 2005, and reforestation has the potential to create more than 34 million hectares of forest by 2020 [4]. In China alone, reforested plantations covered 69.33 million ha in 2014, becoming the largest around the world [27]. Although the influence of reforestation on deep soil OC is still poorly understood [22], deep soil carbon may be influenced by changes in land-use and/or management [28]. The two most frequently used tree species in afforestation in southern China are the coniferous Masson pine (*Pinus massoniana*), and broad-leaved sweet gum (*Liquidambar formosana*) [8]. Both tree species have been widely planted and contribute to reducing soil erosion and providing harvestable wood [8]. The former is dominant during the early stage of forest succession in the mid-subtropics, and the latter during the middle stage [29]. However, the influence of these two vegetation types on OC, especially deep soil OC, is unclear.

In this study, we separated soil aggregates and measured soil OC stability of topsoil and deep soil in the restored forest plantations of subtropical China. Moreover, the relationship between the levels of nitrogen (N) and phosphorus (P) and OC stability at different soil depths was also determined. The goal of this study was to test the following hypotheses: (1) OC is more abundant in topsoil and more stable in deep soil; (2) Soil OC is more stable in coniferous forests (CF) than in broadleaved forest (BF), since the needle litter is less rapidly decomposed and tends to accumulate; (3) Carbon stability decreases with increasing aggregate size irrespective of topsoil or deep soil. Our results will help clarify the influence of vegetation type on OC and nutrient dynamics, and assess the effectiveness of vegetation restoration in the hilly red soil region of subtropical China.

## Materials and Methods

### Study area

This study was conducted in the Vegetation Restoration Ecological Station of Degraded Ecosystem, which lies in a typical red soil hilly area of Taihe County, Jiangxi Province, Southern China (26°44’ N, 115°04’ E). The climate is subtropical with a damp monsoon season, warm, dry summer and a cool, wet winter. Mean annual rainfall is 1600 mm, and 49% of it occurs between April and June. Air temperature ranges from -6°C to 40.7°C (mean annul temperature 18.6°C), and the average temperature is 6.5°C in January (winter) and 29.7°C in July (summer). There are 1306 hours of clear sky per year, and the solar radiation is 4349 MJ m^-2^. The soil is described as Ferralsols (FAO/UNESCO) developed from quaternary red clay, which are moderately well drained and clay textured. The elevation ranges from 75 to 130 m above sea level [8] and the mean slope gradient of this area was about 15°.

The research site had a history of frequent human disturbance (firewood collection) prior to restoration in the 1980s. Prior to this, the site was dominated by secondary shrubs, a disturbed plagioclimax community widely distributed in subtropical regions of China. This site with a 133-ha study area was established by Jiangxi Agricultural University and the local forestry department in 1990 mainly to explore the reforestation effects of silvicultural regimes. Environmental factors such as severe soil erosion and low P availability have made it difficult to establish forest vegetation at this site [30]. The research site did not involve endangered or protected species, and no specific permissions were required for conducting experiment in this site.

### Stand investigation and soil sampling

The stand characteristics of 6 paired plots (20 m × 20 m) in rehabilitated CF and BF distributed in different hills were investigated in July 2011 (Table 1, see S1 Fig). Both plantations were established in late1991 or early 1992. CF was planted with Masson pine at 1.5 m × 2 m spacing. Major understory plants include *Arundinella anomala*, *Cymbopogon citratus*, *Eurya japonica*, *Melastoma dodecandrum*, *Quercus serrata*, *Rosa* spp. and *Viola philippica*. BF was planted with sweetgum (*Liquidambar formosana*) at 1.8 m × 2 m spacing. Understory plants were dominated by *Carex tristachya*, *Dicranopteris dichotoma*, *E*. *japonica*, *Rosa* spp. and *V*. *philippica*.

**Table 1:** Stand characteristics of two restored subtropical plantations in hilly red soil region, southern China.

Stand characteristics	Coniferous forest^a^	Broad-leaved forest^a^
Average density (individuals ha^-1^)	3150±66	3000±88
Diameter at breast height (cm)	10.22±0.25	11.64±0.25
Average height (m)	8.44±0.15	9.84±0.22
Basal area (m^2^ ha^-1^)	25.84±0.01	31.92±0.01
Understory vegetation biomass (t ha^-1^)	8.20±0.30	2.06±0.28
Understory litter biomass (t ha^-1^)	2.50±0.18	3.25±0.14

^a^ Mean±1 standard error, *n* = 6.

We selected 2 locations of two representative trees from each plot at mesoslope in July 2011. At each location we used a steel corer to obtain intact 15 cm × 15 cm × 15 cm soil blocks at 3 depths to 45 cm and four soil samples were obtained in one plot of each soil depth. These blocks were placed in aluminum specimen boxes and transported to laboratory for air-drying. In order to better discriminate the different characteristics in topsoil and deep soil, we define the soil of 0–15 cm layer as “topsoil” and 30–45 cm layer as “deep soil”. Only the samples of these two layers were used for analysis of soil aggregate separation and carbon mineralization. We also collected soil samples using metal cylinder for determining soil bulk density and moisture to 45 cm depth with the same 15 cm interval.

### Soil aggregate separation

Aggregates were separated by dry sieving in order to preserve water-soluble carbon, nutrient and microbial communities within aggregates [31]. Following the procedures established by Kemper and Rosenau (1986)[32], intact soil blocks were crumbled by hand into pieces of approximately 10 mm in diameter. After air-drying, soil samples were sealed in plastic boxes at 4°C in order to preserve the moisture level at which soils can be easily sieved for aggregate fractionation [31]. Briefly, 500 g soil samples were passed through nested sieves with 5-, 2-, 1-, 0.5-, and 0.25-mm mesh. Sieves were placed on a Ro-Tap shaker and shaken at 200 oscillations per min for 5 min. Aggregates retained at each sieving level were air-dried at room temperature and weighed. Mean weight diameter (MWD) of aggregates was calculated by summing the product of mean diameter of aggregates and proportion of total weight for each aggregate size class [32].

### Organic carbon mineralization

The carbon mineralization was evaluated by incubating soil samples from each aggregate size class from topsoil and deep soil. In brief, 20 g soil samples were moistened with distilled water to 50% field capacity and placed in a 500 ml chamber. To ensure an active microbial community, soil samples in incubation chambers were inoculated with a suspension of fresh topsoil from their respective plots. 10 g of fresh soil was mixed with 1000 ml physiological saline and allowed to settle for approximately 6 hours. After adding 1 ml of this supernatant, chambers were incubated for 24 hours, then sealed and incubated at 25°C. Sealed chambers contained alkali CO_2_ traps consisting of 5 ml 2 M NaOH in 10 ml beakers. The CO_2_ traps were changed on days 5, 10, 15, 22, 32, 43, 57 and 71, and the OC mineralization (CO_2_ emission) was measured by titration with standardized 0.5 M HCl after precipitation of CO_2_ with the addition of 1 M BaCl_2_ [33]. Soil OC mineralization of aggregates was calculated based on the soil dry weight (cumulative carbon mineralization, C_min_, g CO_2_–C kg^−1^ soil) and soil OC concentration (SOC mineralized, SOC_min_, % SOC), respectively.

In order to compare soil OC mineralization of various soil aggregate sizes, the single first-order model of *C*_*m*_ = *C*_*0*_ *(1-e*^*-kt*^*)* was used. *C*_*m*_ is the cumulative percentage of SOC mineralized (% SOC) during incubation, *C*_*0*_ is potential percentage of soil OC mineralized (% SOC), which represents the proportion to the amount of available carbon during soil OC mineralized at a given time [34], *k* and *t* represent the mineralization constant and duration of incubation (days), and days required for decomposition of half of mineralizable carbon (*t*_*0*.*5*_) is calculated by ln2/k, respectively.

### Soil analysis

Soil bulk density was determined using a 100 cm^3^ metal cylinder. Soil moisture was calculated gravimetrically by drying soils at 105°C overnight and the water content was expressed as a percentage of the dry weight. Soil pH was measured in a 1:2.5 mixture of soil and deionized water using a glass electrode. All soil samples were oven-dried and sieved with a 0.15 mm screen prior to nutrient measurement. Organic C was determined using the Walkley-Black wet oxidation method [35]. Soil total N and total P concentrations were determined by the Kjeldahl method and by the molybdenum-stibium colorimetric method, respectively, after samples were digested with 1.84 M H_2_SO_4_[35]. The nutrient stock in bulk soil was calculated based on the concentration and bulk density.

### Statistical analysis

All statistical analyses were conducted using SPSS 16.0. All data were tested for homogeneity of variance (Levene’s test) before statistical analysis. The CF and BF, and topsoil and deep soil characteristics were compared by paired *T* tests. One-way ANOVA and least significant difference (LSD) method were used to compare the differences of OC, TN, TP and OC mineralization parameters among six aggregate sizes. The standard 0.05 level was used throughout as a cutoff for statistical significance.

## Results

### General soil characteristics and aggregate distribution

Soil bulk density, soil moisture and pH did not differ significantly between soil sampled from CF and BF at any depth (Table 2). The OC concentration, total N and C/P were higher in BF than in CF, and decreased with increasing soil depths in both forests. Total P concentration and P stocks were much higher in BF topsoil than at other depths in both forests. Soil C/N was the highest at the 15–30 cm depth in CF and the lowest in BF deep soil. Soil N/P was the highest in CF topsoil, followed by the three soil depths in BF, and lowest in CF deep soil samples (Table 2).

**Table 2:** Soil physical-chemical properties of two restored subtropical plantations in hilly red soil region, southern China.

Properties	Coniferous forest^a^			Broad-leaved forest^a^		
	0–15 cm	15–30 cm	30–45 cm	0–15 cm	15–30 cm	30–45 cm
Bulk density (g cm^-3^)	1.63±0.05a	1.66±0.03a	1.68±0.06a	1.71±0.03a	1.65±0.04a	1.71±0.04a
Mean weight diameter (MWD, mm)	3.04±0.51b	/	3.13±0.33b	4.19±0.14a	/	4.45±0.33a
Moisture (%)	12.17±0.61a	12.86±0.73a	12.22±0.55a	13.28±0.43a	12.99±0.67a	13.40±0.59a
pH	4.52±0.02a	4.42±0.02a	4.53±0.01a	4.53±0.03a	4.46±0.04a	4.59±0.05a
Organic C concentration (g kg^-1^)	5.18±1.00a	2.37±0.41b	1.05±0.17c	6.64±0.56a	2.97±0.17b	1.40±0.03c
Total N concentration (g kg^-1^)	1.10±0.04b	0.34±0.05d	0.39±0.10d	1.50±0.29a	0.72±0.04c	0.46±0.03d
Total P concentration (g kg^-1^)	0.26±0.05b	0.36±0.03b	0.35±0.02b	0.53±0.03a	0.33±0.06b	0.23±0.03b
C/N	4.86±1.04b	7.13±0.67a	4.46±1.43b	4.92±0.64b	4.21±0.28b	3.11±0.23c
C/P	27.96±8.73a	6.88±1.50c	2.99±0.46d	12.90±1.40b	10.65±1.67b	6.78±0.86c
N/P	5.71±1.60a	0.98±0.20c	1.12±0.30c	2.83±0.49b	2.61±0.43b	2.22±0.30b
C stock (t ha^-1^)	12.90±2.75b	5.90±1.04c	2.70±0.48d	16.98±1.36a	7.35±0.42c	3.58±0.10d
N stock (t ha^-1^)	2.67±0.08b	0.85±0.14d	0.97±0.26d	3.87±0.78a	1.77±0.10c	1.18±0.08d
P stock (t ha^-1^)	0.63±0.12bc	0.90±0.07b	0.88±0.06b	1.36±0.10a	0.79±0.15bc	0.58±0.08c

^a^ Mean±1 standard error, *n* = 6;

Different letters indicate the differences among three depths in two plantations.

**Fig 1:**
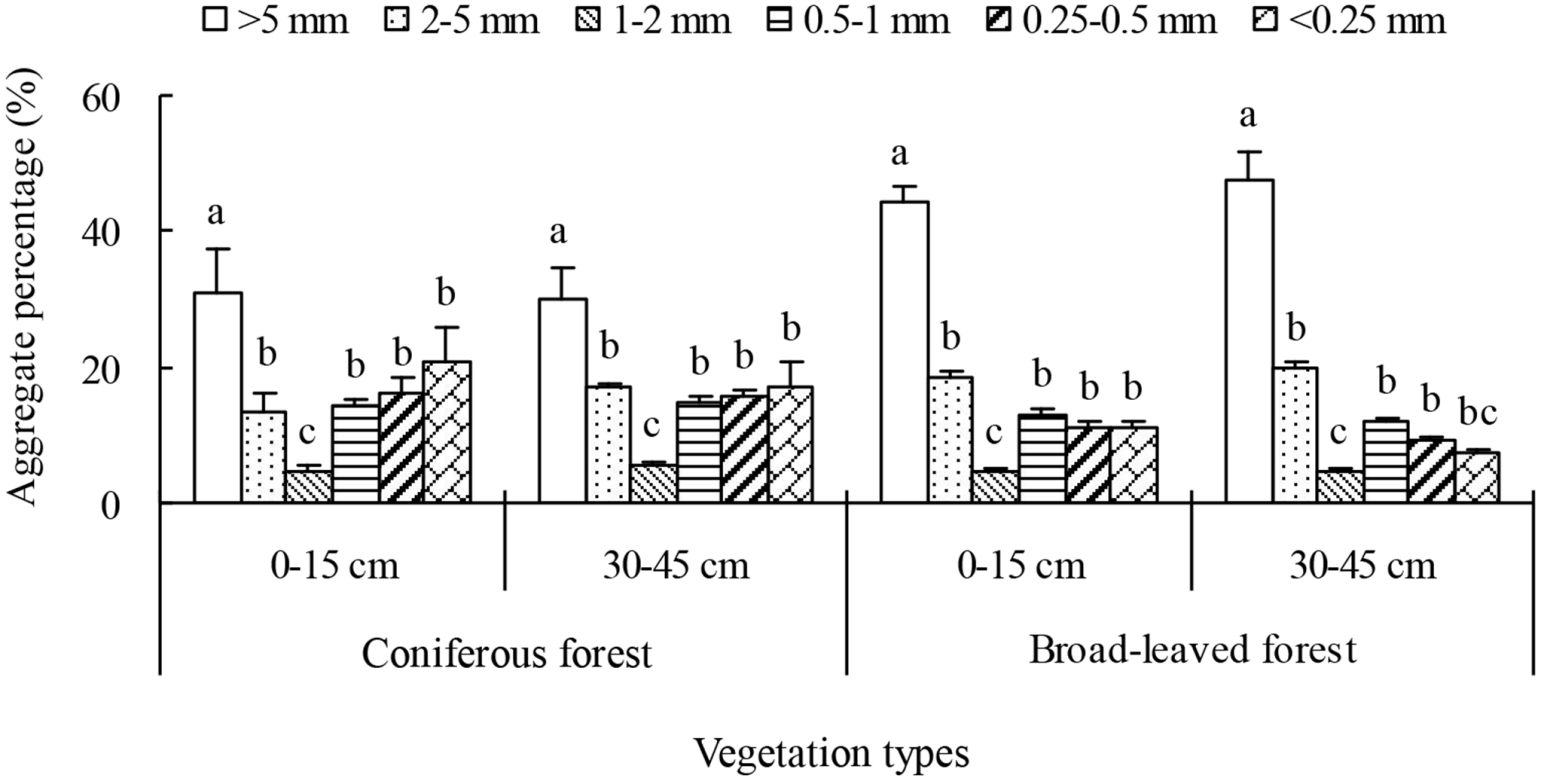
The components of soil aggregate fractions of two depths in two restored plantations of subtropical China. The legend means the aggregate diameter. Error bars show the standard error of the mean. The different letters represent significance differences among the different soil aggregate fractions within a depth (*P*<0.05).

The mass of soil aggregates of >5 mm diameter was the greatest followed by 2–5 mm, 0.5–1 mm, 0.25–0.5 mm, and <0.25 mm, and that of 1–2 mm aggregates was the lowest (Fig 1). Meanwhile, MWD was higher in BF than in CF samples, but did not differ significantly between topsoil and deep soil in each forest type (Table 2).

### Organic carbon concentration and mineralization in aggregates of different sizes

**Fig 2:**
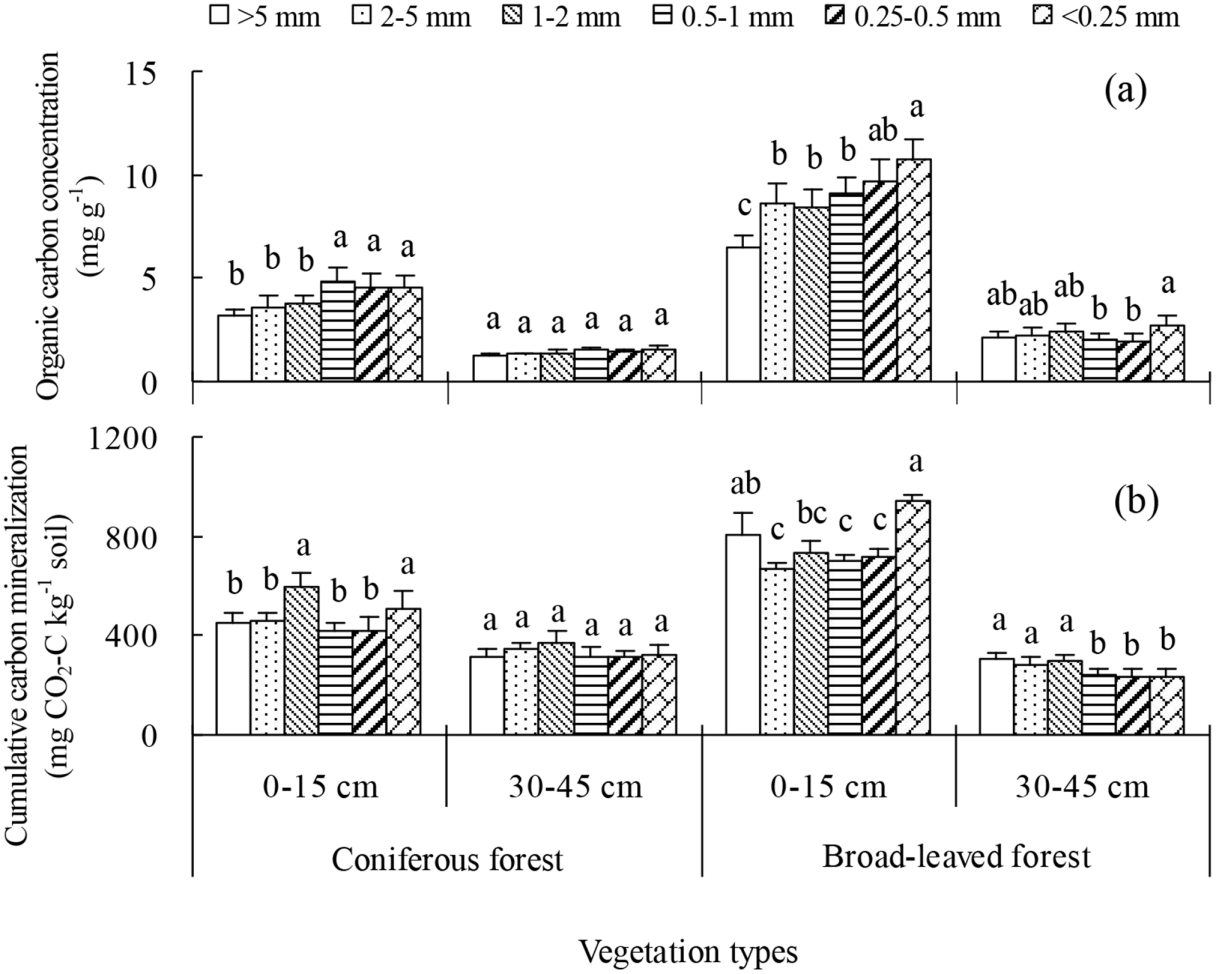
The organic carbon concentration and mineralization of aggregate soil within 71 days at various soil depths in two restored plantations of subtropical China. Error bars show the standard error of the mean. The different letters represent significance differences among the different soil aggregate fractions within a depth at *P*<0.05 level.

Smaller aggregates had a higher OC concentration (0.5–1 mm, 0.25–0.5 mm and <0.25 mm) than larger aggregates (>5 mm, 2–5 mm and 1–2 mm) in CF topsoil, and OC concentration decreased with increasing aggregate size in BF topsoil. In contrast, the OC concentration varied very little between aggregate size classes at deep soils in both forests (Fig 2a).

The C_min_ during the first 15 days was the highest in aggregates of 1–2 mm and <0.25 mm, followed by >5 mm and 2–5 mm, and the lowest in aggregates of 0.5–1 mm and 0.25–0.5 mm in CF topsoil (S2a Fig). Similarly in BF topsoil, the C_min_ during the first 15 days was higher in <0.25 mm aggregates than in other aggregates, and did not differ significantly between the six aggregate categories at deeper soil depths in either vegetation type (S2a Fig).

In CF topsoil, the C_min_ measured over 43 and 71 days were generally higher in aggregates of 1–2 mm and <0.25 mm than in other aggregates, but such patterns were not observed in deep soil. In BF topsoil, the C_min_ measured over 43 and 71 days were generally higher in aggregates of >5 mm and <0.25 mm than in other aggregates, and higher in larger aggregates (>5 mm, 2–5 mm and 1–2 mm) than in smaller aggregates (0.5–1 mm, 0.25–0.5 mm and <0.25 mm) in deep soils (Fig 2b and S2b Fig).

In general, the SOC_min_ trended to be higher in larger aggregates (>5 mm, 2–5 mm and 1–2 mm) than in smaller aggregates (0.5–1 mm, 0.25–0.5 mm and <0.25 mm) in two soil depths under two forest types (Table 3). In CF topsoil, the SOC_min_ was significantly higher in aggregates of 1–2 mm than that in aggregates of 0.5–1 mm and 0.25–0.5 mm, while the highest value of OC mineralization percentage was found in aggregates of >5mm in BF topsoil (Table 3).

**Table 3:** The percentage of soil organic carbon mineralized in various aggregates within 71 incubation days in two soil depths under two restored plantations of subtropical China.

Aggregates (mm)	Coniferous forest^a^		Broad-leaved forest^a^	
	0–15 cm	30–45 cm	0–15 cm	30–45 cm
>5	2.60±0.22ab	4.52±0.42a	2.26±0.20a	2.83±0.41a
2–5	2.57±0.38ab	4.74±0.48a	1.49±0.20b	2.36±0.24a
1–2	3.25±0.75a	4.86±0.59a	1.64±0.16b	2.49±0.40a
0.5–1	1.75±0.29b	3.76±0.54a	1.44±0.12b	2.44±0.41a
0.25–0.5	1.83±0.32b	4.63±1.01a	1.41±0.15b	2.48±0.56a
<0.25	2.17±0.39ab	3.86±0.59a	1.62±0.12b	1.79±0.35a
Weighted mean	2.32±0.3B	4.59±0.37A	1.76±0.15B	2.49±0.25B

^a^ Value = mean±1 standard error, *n* = 6;

Different lowercase letters represent significance differences among the different soil aggregate fractions within a depth and different uppercase letters indicate the differences among two depths in two plantations.

**Fig 3:**
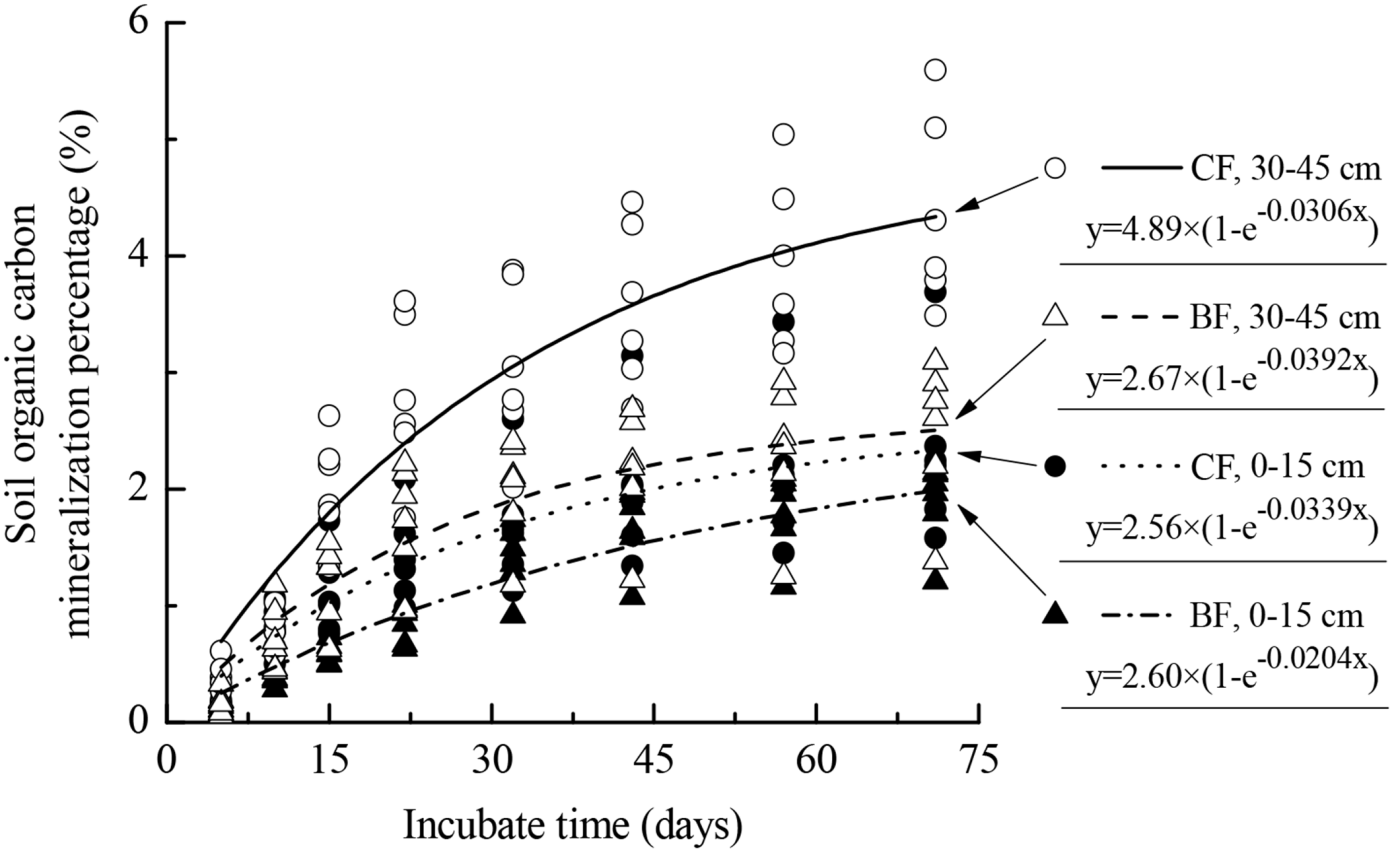
The weighted mean of soil organic carbon mineralized percentage in various aggregates vary with incubation days in two soil depths under two restored plantations of subtropical China. CF and BF indicate coniferous forest and broad-leaved forest, respectively. Organic carbon mineralization modeling *C*_*m*_ = *C*_*0*_ *(1-e*^*-kt*^*)*. *C*_*m*_ and *C*_*0*_ indicates the accumulative amount of organic carbon mineralization percentage within the incubation days and potential mineralization percentage, respectively; *k* and *t* indicate the mineralization constant and days, respectively.

Likewise, the soil OC mineralized potential (*C*_*0*_), mineralization constant (*k*) and decomposition days of half mineralizable carbon (*t*_*0*.*5*_) varied with aggregate size, vegetation type and soil depth (Table 4). The *C*_*0*_ was higher in CF than in BF soil aggregates at both depths, while the *t*_0.5_ in BF topsoil aggregates exceeded those in topsoil aggregates of CF (Table 4). In CF, the *C*_*0*_ and *t*_0.5_ were higher in deep soil aggregates than in topsoil aggregates, however, the *t*_0.5_ was lower in deep soil aggregates than in topsoil aggregates in BF (Table 4 and Fig 3). Additionally, the percentage of *C*_*m*_ to *C*_*0*_ reached roughly 50% on day 15, and 85% on day 43, of the mineralization achieved by 71 days (Table 4 and Fig 3).

**Table 4:** The parameters of organic carbon mineralization kinetic model in two restored subtropical plantations of China.

Aggregates (mm)	Coniferous forest^a^				Broad-leaved forest^a^			
	*C*_0_ (% SOC)	*k* (day^-1^)	*r*	*t*_0.5_^b^ (day)	*C*_0_ (% SOC)	*k* (day^-1^)	*r*	*t*_0.5_^b^ (day)
0–15 cm								
>5	2.82	0.0372	0.97	18.6	3.32	0.0182	0.95	38.2
2–5	2.88	0.0328	0.97	21.1	1.94	0.0227	0.97	30.5
1–2	3.69	0.0313	0.97	22.2	2.28	0.0202	0.96	34.4
0.5–1	1.95	0.0322	0.98	21.5	1.94	0.0211	0.97	32.8
0.25–0.5	1.99	0.0339	0.97	20.4	1.95	0.0203	0.96	34.2
<0.25	2.34	0.0376	0.96	18.4	1.93	0.0299	0.94	23.2
30–45 cm								
>5	5.28	0.0270	0.96	25.7	3.03	0.0393	0.96	17.6
2–5	5.35	0.0316	0.94	21.9	2.58	0.0377	0.95	18.4
1–2	5.23	0.0354	0.96	19.6	2.63	0.0440	0.95	15.8
0.5–1	4.12	0.0318	0.94	21.8	2.55	0.0455	0.94	15.2
0.25–0.5	5.13	0.0326	0.95	21.2	2.68	0.0355	0.95	19.5
<0.25	4.21	0.0332	0.96	20.9	1.88	0.0425	0.96	16.3

^a^ Calculated with the formula *C*_*m*_ = *C*_*0*_ *(1-e*^*-kt*^*)*. *C*_*m*_ and *C*_*0*_ indicate the cumulative percentage of soil organic carbon mineralized within the incubation days and potential percentage of soil organic carbon mineralized, respectively; *k* and *t* indicate the mineralization constant and days, respectively.

^b^ *t*_0.5_ indicates the required days for attaining half potential percentage of organic carbon mineralized.

### Nutrient concentration and carbon stability relationship in soil aggregates

**Fig 4:**
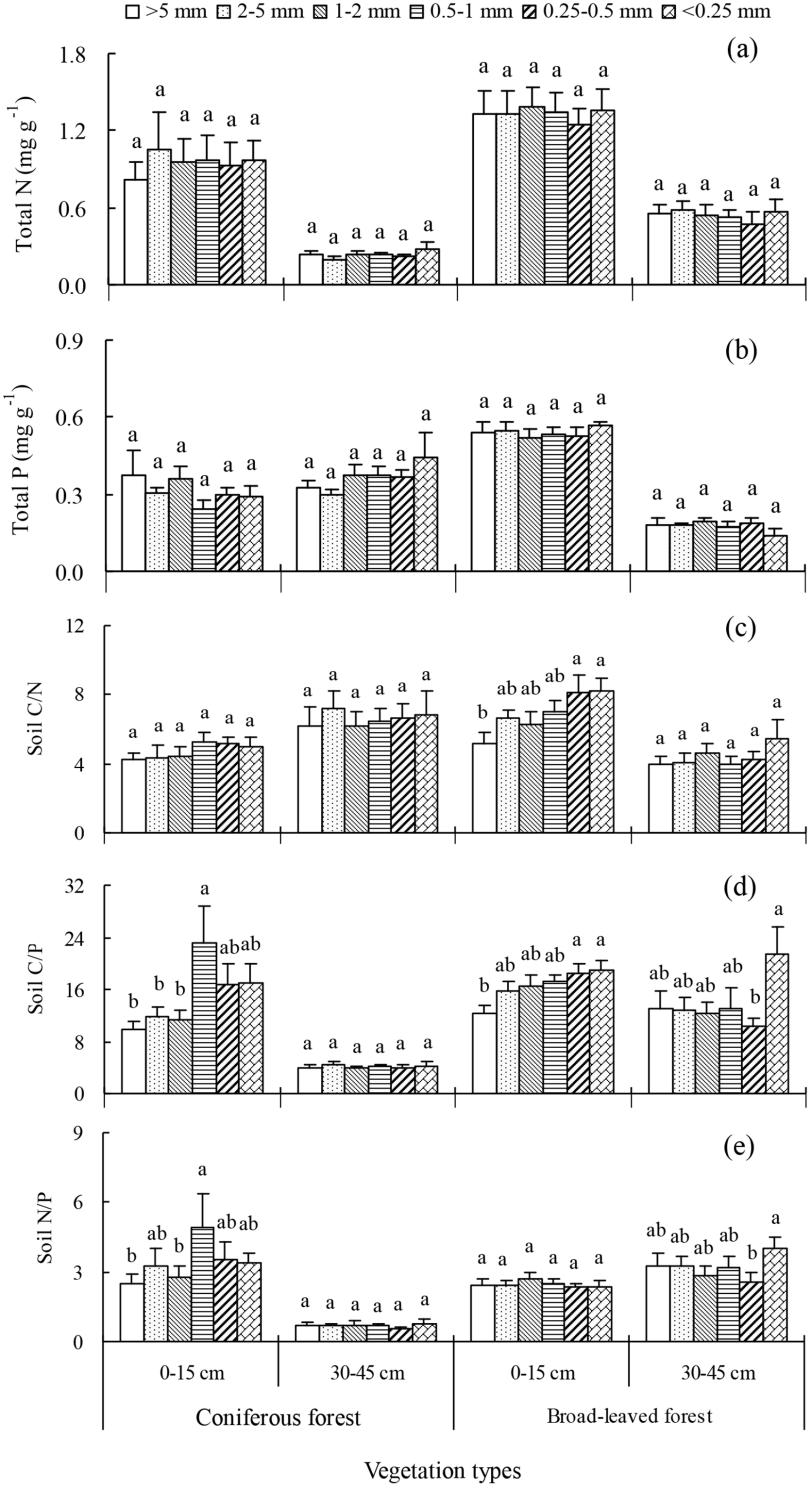
The concentrations of total nitrogen and phosphorous in various aggregates varied with soil depth in two restored plantations of subtropical China. Error bars show the standard error of the mean. The different letters represent significance differences among the different soil aggregate fractions within a depth (*P*<0.05).

The total soil N and P concentrations did not differ significantly between the six soil aggregate sizes at any depth or vegetation type (Fig 4a and 4b). However, total N was generally higher in BF than in CF and much higher in topsoil than in deep soil at each aggregate (Fig 4a). CF topsoil aggregates contained less P than BF topsoil aggregates, but CF deep soil aggregates contained more P than BF deep soil aggregates. Additionally, the total P concentration was higher in topsoil aggregates than in deep soil aggregates in BF, but did not differ significantly between these depths in CF (Fig 4b).

Soil C/N varied with aggregate size in BF topsoil, where it generally decreased with increasing aggregate size (Fig 4c). Soil C/P was higher in smaller than larger aggregates in topsoil of two vegetation types, and was the highest in aggregates under <0.25 mm in CF (Fig 4d). Soil N/P was higher in smaller than larger aggregates in CF topsoil, however, this pattern of distribution was not found in BF topsoil. In deep soil, soil N/P was higher in BF than in CF at any aggregate size (Fig 4e).

Meanwhile, C_min_ generally correlated positively with total OC, total N, total P, C/N and C/P (Table 5 and S1 Table). But C_min_ negatively correlated with C/N in CF. Moreover, C_min_ correlated positively with OC and total N in topsoil, but negatively in deep soil. The SOC_min_ during the incubation period generally negatively correlated with OC, total N, total P, C/N, C/P and N/P. In contrast, total P positively correlated with the SOC_min_ in deep soil, but not in topsoil (Table 5 and S1 Table).

**Table 5:** Correlation co-efficiencies among soil aggregate nutrients vs. organic carbon mineralization of 71 days in two restored subtropical plantations of China.

Variables	TOC	TN	TP	C/N	C/P	N/P
Cumulative carbon mineralization, C_min_^a^						
All data (*n* = 144)	0.78***	0.67***	0.68***	0.27**	0.28**	0.04^NS^
Coniferous forest(*n* = 72)	0.51***	0.52***	0.09^NS^	-0.33**	0.24*	0.29*
Broad-leaved forest (*n* = 72)	0.83***	0.75***	0.88***	0.58***	0.23*	-0.35**
0–15 cm (*n* = 72)	0.63***	0.41***	0.64***	0.42***	0.00^NS^	-0.30**
30–45 cm (*n* = 72)	-0.72***	-0.61***	0.40***	0.07^NS^	-0.63***	-0.62***
Soil organic carbon mineralized, SOC_min_^a^						
All data (*n* = 144)	-0.62***	-0.62***	-0.14^NS^	-0.07^NS^	-0.68***	-0.56***
Coniferous forest(*n* = 72)	-0.73***	-0.62***	0.10^NS^	0.12^NS^	-0.64***	-0.61***
Broad-leaved forest (*n* = 72)	-0.63***	-0.54***	-0.46***	-0.48***	-0.61***	-0.15^NS^
0–15 cm (*n* = 72)	-0.65***	-0.54***	-0.30*	-0.25*	-0.58***	-0.32**
30–45 cm (*n* = 72)	-0.72***	-0.61***	0.40**	0.07^NS^	-0.63***	-0.62***

^aNS^ not significant,

* *P*<0.05,

** *P*<0.01,

***P<0.001.

## Discussion

### Deep soil organic carbon stock influenced by vegetation type

The stability of deep soil carbon stocks has received increased attention in recent years [12,13,14]. In this study, the soil OC concentration and OC stock in BF soil were greater than that in CF soil at any soil depth. Moreover, the absolute difference in soil OC stock between topsoil and deep soil in BF (13.40 t ha^-1^) was higher than that in CF (10.20 t ha^-1^) (Table 2). Therefore, the influence on deep soil OC stock varied with vegetation type. Various mechanisms by which OC stocks in deep soils are increased following reforestation have previously been described [9,11,12]. The characteristics of deep soil OC are mainly influenced by three key processes, including the input of organic matter from biological residuals (roots, root exudates, soil animals and microorganisms), OC transportation into deep soil from topsoil, and physical protection and decomposition of organic matter [28].

Root biomass accumulation and input through root turnover was considered as an important factor in carbon sequestration following reforestation [36,37]. In the present study, we did not directly measure the root biomass, however a previous publication reported a study of the same climatic zone and similar soil condition [38]. Analysis of the 21-year old Masson pine and sweet gum plantations indicated that the annual average biomass of living and dead fine roots in the pine plantation was higher than that in the sweet gum plantation at 30–45 cm soil depth [38]. However, the value of fine root biomass at 0–15 cm soil profile was higher in sweet gum plantation than in Masson pine plantation [38]. Dead roots could supply OC to the soil by microbial conversion. Contrary to expectation, the potential higher root input in CF did not lead to a greater OC stock in deep soils, in comparison with BF. We speculate that the transfer of OC from topsoil to deep soil, originating from fine root decomposition, may be an important source of deep soil OC stocks in BF.

In addition to the direct input of carbon from roots at depth, deep soil carbon may also come from the vertical transport of dissolved OC [12,39]. The movement and retention of dissolved OC within mineral soil was found to be responsible for 20% of the total mineral soil carbon stock to a depth of 1 m in a Californian forest soil [40]. Previous research had found that the dissolved OC concentration in BF was significantly higher than that in CF at 0–10 cm and 10–20 cm soil layer of same study location [41]. In our study, BF topsoil carbon stock exceeded CF carbon stock. Therefore, we deduced that transport of dissolved OC from the topsoil to depth was greater in BF than in CF, increasing the deep BF OC stock despite a lower root biomass input. Moreover, in this study, the percentage of soil OC mineralization of aggregates in deep soils was higher in CF than in BF (Table 3), implying there might be higher OC decomposition in CF deep soils compared with that in BF deep soils.

Finally, we found the regulation of carbon cycling in CF and BF between the topsoil and deep soil was different. In this study, OC was much lower in deep soil than topsoil in CF, and also lower in CF than in BF. However, the C_min_ occurring within 15–71 days did not differ significantly between deep soil and topsoil in CF (Fig 2b and S2b Fig). Therefore, we deduced that the deep soil may be more readily decomposed in CF than in BF, potentially as a result of a higher dead fine root biomass, since fresh carbon may accelerate soil OC decomposition [10]. To sum up, organic matter decomposition and OC transportation from topsoil to deep soil might be the dominant processes influencing deep soil OC in these soils.

### Soil organic carbon stability influenced by aggregate size and tree species

The stability of soil OC was influenced by soil aggregation, providing microenvironments of physical protection and absorbing particle organic matter [7,23]. The SOC_min_ trended to be higher in larger aggregates than in smaller aggregates in two soil depth under two forest types (Table 3). Similarly, the C_min_ was greatest in larger aggregates (>1 mm) at two soil depth in CF and was greater in larger than smaller aggregates at BF deep soil (Fig 2b). In general, the protection degree of soil OC is different in varied aggregates classes [7,42]. Previous studies had shown that the degree of protection to microaggregates on OC was higher than that of the macroaggregates, since the mean residence time (MRT) of OC in macroaggregates is less than in microaggregates [43,44,45]. von Lützow et al.[17] reported that the turnover time of OC in macroaggregates and microaggregates was 15–50 years and as long as 100–300 years using ^13^C natural abundance method, respectively, which indicates that microaggregates are more effective for decreasing OC mineralization relative to macroaggregates. These conclusions were basically supported by our findings, that the value of SOC_min_ was higher in larger aggregates than in smaller aggregates at topsoils and deep soils of two forest types. In this study, we also found higher OC concentration in topsoil smaller aggregates, probably because smaller aggregates have larger surface area and then can absorb more OC. On the other hand, decomposition efficiency of microbe and enzyme on OC may be lower in smaller aggregates owing to greater physical protection, in the light of the theory that microaggregate is formed within macroaggregate structure [23].

Generally, physical protection is one of the important mechanism to carbon stability. Compared with BF, CF had more smaller soil aggregates and fewer larger soil aggregates, and the MWD was lower in CF than that in BF deep soils, which means the stability of the soil OC was better in CF [46]. However, the value of SOC_min_ was significantly higher in CF than in BF and there was no difference of C_min_ in deep soil of CF and BF (Fig 2 and Table 3). Soil organic matters were the adhesive in the formation of soil aggregates [6], which mainly came from root exudates and decomposition of microbes on plant residue [13]. Soil aggregates might not be a major factor controlling OC stability when soil OC concentration was both low both in CF and BF at the early stages of vegetation restoration (21yr). Thus SOC_min_ was not lower in CF with relatively higher percentage of smaller soil aggregates than in BF. We speculate that the aggregate protective effects on carbon will appear more intense with the advance of the recovery process.

Therefore, the soil OC stability in different forest types may be determined by other factors [47,48]. In this study, we found positive correlation between SOC_min_ and total P concentration and negative correlation between SOC_min_ and total N, C/P, N/P in deep soil (Table 5). It has been reported that the increase of N may inhibit soil respiration in tropical and subtropical regions, while the increase of P would promote the cycle of C [49]. Our previous study also reported that P enrichment can degrade the stability of OC in urban vegetation soil because P increases microbial activity on soil protected C [50]. That response likely arises because N is more closely coupled to organic matter cycling, and P is limiting element in ecosystem [49,51]. In fact, soil OC mineralization is the process of microbial participation in the release of CO_2_ [52]. Due to the different stoichiometric ratios between microorganism and the substrate, the microbes will have different OC utilization efficiencies under different stoichiometric ratios of substrate for nutrient requirement [53,54], which may induce different OC mineralization. Manzoni et al. [52]considered that stoichiometry controlled microbial carbonuse efficiency in soils and Buchkowski et al. [55] reported that microbial stoichiometry overrode biomass as a regulator of soil carbon cycling. Therefore, the higher SOC_min_ in CF than in BF in deep soil may be induced by the differences of nutrient concentration, C/P and N/P between two vegetation types, since P concentration was higher in CF and broadleaf litters with high N/P ratios accumulated in the BF [56].

It has been found that deep soil OC has old radiocarbon age, which suggests that deep soil OC is stable on longer time scales [57,58]. In general, root litter and exudates were the main resources contributing to deeper soil OC input [36], which may be chemically more recalcitrant than topsoil litter because of higher concentrations of aliphatic and lignin material [59]. Moreover, acid hydrolysis process in soil was considered to remove easily decomposable protein and polysaccharide material leaving behind chemical recalcitrant structures [13], which may be able to isolate deeper soil C with long-term stability due to the evidence that the C isolated by acid hydrolysis from deeper soil was several hundred or thousand years older than bulk soil [60]. However, the SOC_min_ of deep soil was greater than that of topsoil in this study, whether in CF or in BF. Soil microbial biomass and its activity were the principal driver regulating the C cycle and stability [13]. Taylor et al. [61] considered that deep soil was metabolically active and contained substantial numbers of microorganisms despite the low biomass contents, which was consistent with the finding that deep soils had a higher value of C_mic_/C_org_ quotient (ratio of microbial biomass carbon to OC) [62]. Similarly, Blume et al. [63] reported that microbial activity in deep soil was similar to that measured in topsoil when normalized to biomass size. Therefore, it would not be surprised that deep soil had a higher SOC_min_ in view of lower OC concentration compared with topsoil. However, this study was carried out under controlled laboratory conditions, and the relevant works on deep soil OC stability at the field scale yet need to be demonstrated in order to compare the results with laboratory studies.

**Fig 5:**
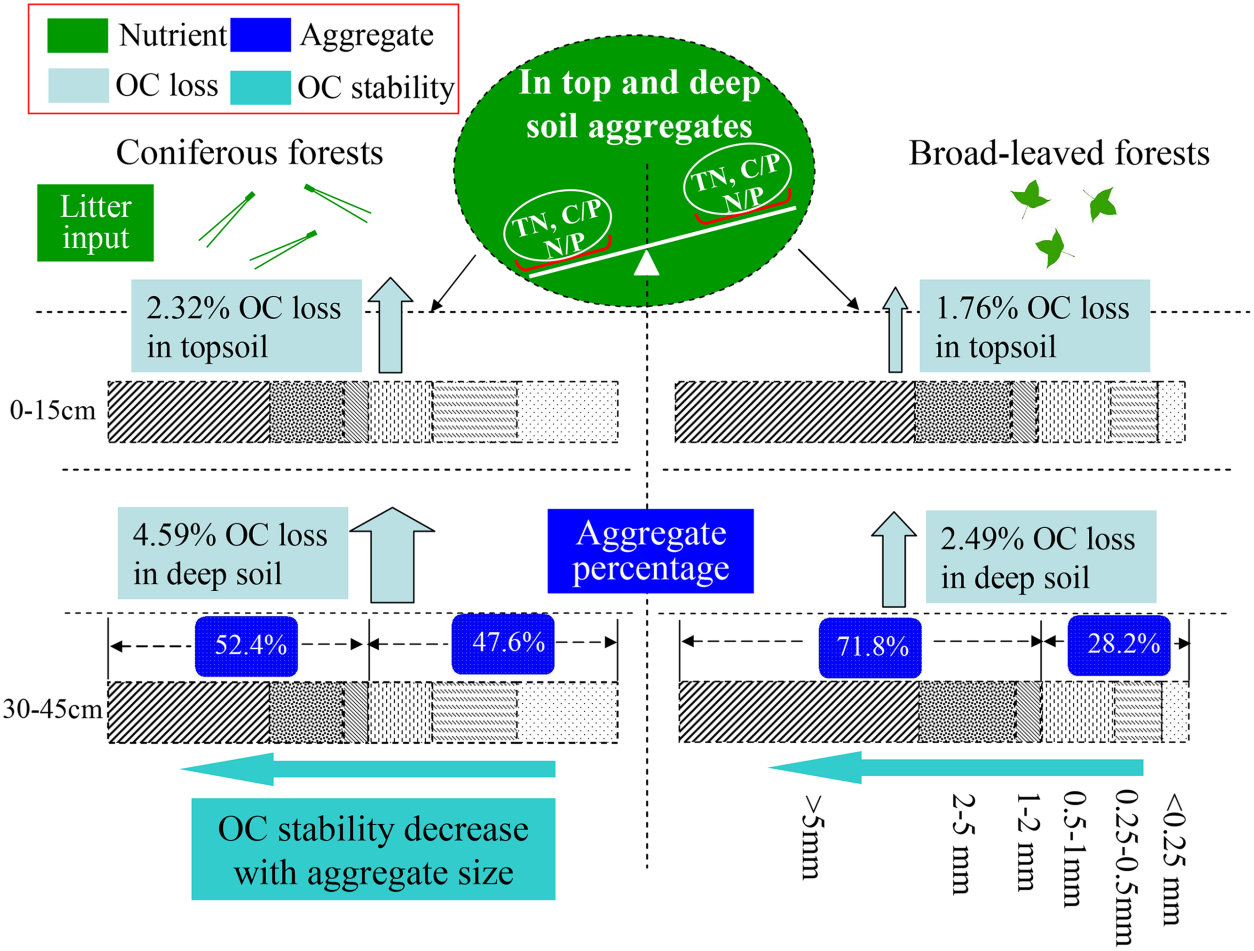
A stylized illustration of the mechanical framework shows the difference of OC stability influenced by nutrient concentration and aggregate composition in two restored plantations. The up arrow represents the OC loss percentage and the width of the up arrow indicates the relative mineralization percentage of OC in topsoil and deep soil aggregates. The left arrow show the direction of OC stability decreased with aggregate size in topsoil and deep soil.

Clearly, reforestation tree species appeared to be an important determinant of OC stability through the influence on soil nutrient and its stoichiometric ratio [30] and BF might be more efficient in OC conservation than CF at the sites we studied (Fig 5) and deep soils may have lower OC stability than topsoil.

## Conclusions

We found vegetation type (coniferous vs. broad-leaved forest) specific effects on soil aggregate formation and nutrient accumulation in the degraded site. Soil OC concentration and storage were higher in BF than in CF at topsoil and deep soil. Soil OC stability trended to be higher in smaller soil aggregates than in larger soil aggregates, while OC stability between CF and BF was dominated by the differences of N, P concentrations and their stoichiometry in two reforested vegetation types. Compared with topsoil, deep soil had lower soil OC stability and the highest value of soil OC mineralized was found in CF deep soils. Thus, soil depth and aggregate size were the important factors influencing soil OC stability, and reforested vegetation type might play an important role in soil OC storage by affecting ecosystem nutrient (especially N and P) cycling. Our results highlight that nutrient alteration shall be the primary concern during vegetation restoration, since nutrient is the key factor to dominate OC dynamics and assess soil quality [64]. A long-term monitoring focusing on interaction among soil physical, chemical and biological properties needs to be developed in order to validate our findings.

## Supporting Information

S1 Fig The location of selected plots [6 replications for coniferous plantations (CF Δ) and broad-leaved forests (BF ○)]. Source from the original drawing of afforestation design in College of Forestry, Jiangxi Agricultural University. The contour interval is 2.5 m.
(TIF)

S2 Fig The cumulative carbon mineralization of aggregate soil within 15 and 43 days at various soil depths in two restored plantations of subtropical China. Error bars show the standard error of the mean. The different letters represent significance differences among the different soil aggregate fractions within a depth at *P*<0.05 level.
(TIF)

S1 Table Correlation co-efficiencies among soil aggregate nutrients vs. organic carbon mineralization of 15 and 43 days in two restored subtropical plantations of China. (DOC)
