# Supplementary figures and images for: Topsoil and Deep Soil Organic Carbon Concentration and Stability Vary with Aggregate Size and Vegetation Type in Subtropical China
Source: PLoS One. 2015 Sep 29;10(9):e0139380. doi: 10.1371/journal.pone.0139380 (PMC5457303; doi:10.1371/journal.pone.0139380)

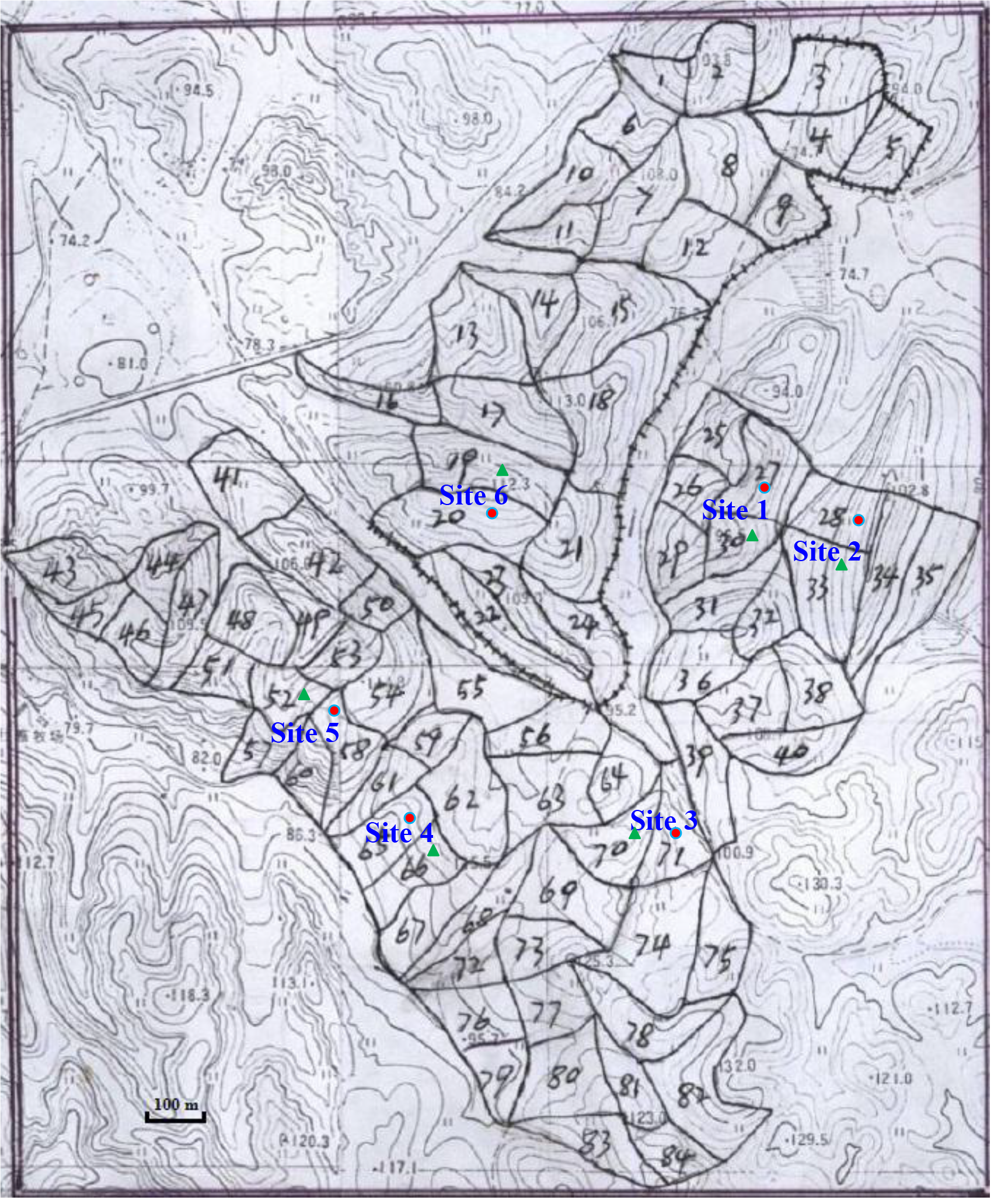

Supplement: S1 Fig — Source from the original drawing of afforestation design in College of Forestry, Jiangxi Agricultural University. The contour interval is 2.5 m. (TIF) [file pone.0139380.s001.tif]

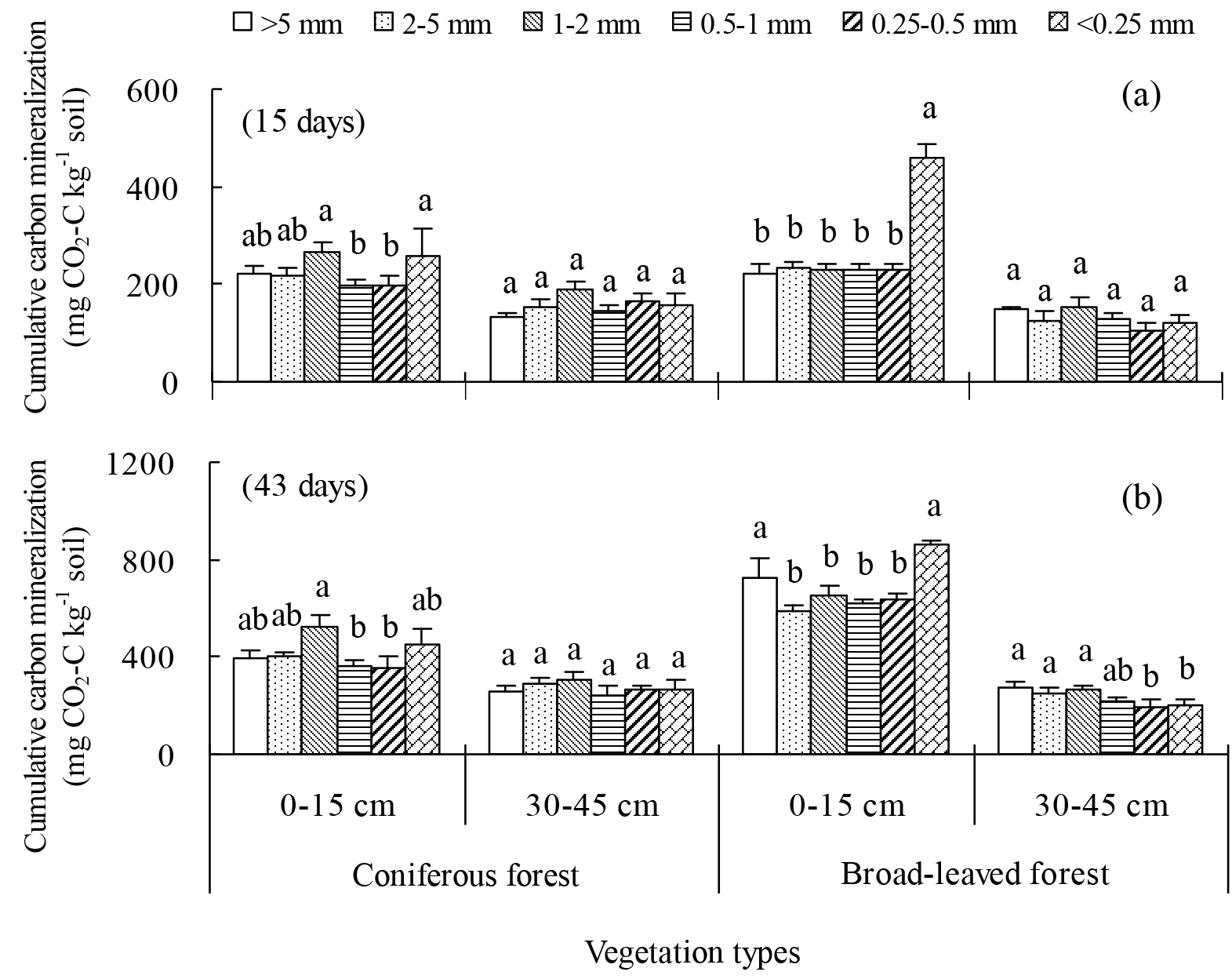

Supplement: S2 Fig — Error bars show the standard error of the mean. The different letters represent significance differences among the different soil aggregate fractions within a depth at P<0.05 level. (TIF) [file pone.0139380.s002.tif]
