# Supplementary material for: Topsoil and Deep Soil Organic Carbon Concentration and Stability Vary with Aggregate Size and Vegetation Type in Subtropical China
Source: PLoS One. 2015 Sep 29;10(9):e0139380. doi: 10.1371/journal.pone.0139380 (PMC5457303; doi:10.1371/journal.pone.0139380)
Supplement: S1 Table — (DOC) [file pone.0139380.s003.doc]

**S1 Table. Correlation co-efficiencies among soil aggregate nutrients vs. organic carbon mineralization of 15 and 43 days in two restored subtropical plantations of China.**

| Variables | Days | TOC | TN | TP | C/N | C/P | N/P |
| --- | --- | --- | --- | --- | --- | --- | --- |
| Cumulative carbon mineralization, C_min_^a^ | | | | | | | |
| All data (*n*=144) | 15 | 0.64*** | 0.54*** | 0.52*** | 0.20* | 0.24** | 0.66*^NS^* |
|  | 43 | 0.79*** | 0.69*** | 0.67**** | 0.24** | 0.30*** | 0.07*^NS^* |
| Coniferous forest (*n*=72) | 15 | 0.47*** | 0.47*** | 0.06*^NS^* | -0.28* | 0.26* | 0.30* |
|  | 43 | 0.53*** | 0.55*** | 0.08*^NS^* | -0.39** | 0.27* | 0.33** |
| Broad-leaved forest (*n*=72) | 15 | 0.78*** | 0.64*** | 0.72*** | 0.51*** | 0.25* | -0.25* |
|  | 43 | 0.83*** | 0.76*** | 0.87*** | 0.57*** | 0.23* | -0.34** |
| 0-15 cm (*n*=72) | 15 | 0.46*** | 0.28* | 0.34** | 0.23* | 0.08*^NS^* | -0.12*^NS^* |
|  | 43 | 0.64*** | 0.42*** | 0.65*** | 0.41*** | 0.02*^NS^* | -0.28* |
| 30-45 cm (*n*=72) | 15 | -0.74*** | -0.57*** | 0.39*** | 0.07*^NS^* | -0.64*** | -0.59*** |
|  | 43 | -0.70*** | -0.56*** | 0.37** | 0.01*^NS^* | -0.60*** | -0.58*** |
| Soil organic carbon mineralized, SOC_min_^a^ | | | | | | | |
| All data (*n*=144) | 15 | -0.68*** | -0.66*** | -0.23** | -0.11*^NS^* | -0.67*** | -0.50*** |
|  | 43 | -0.60*** | -0.59*** | -0.13*^NS^* | -0.11*^NS^* | -0.66*** | -0.53*** |
| Coniferous forest (*n*=72) | 15 | -0.74*** | -0.64*** | 0.10*^NS^* | 0.16*^NS^* | -0.63*** | -0.61*** |
|  | 43 | -0.69*** | -0.58**** | 0.10*^NS^* | 0.06*^NS^* | -0.61*** | -0.57*** |
| Broad-leaved forest (*n*=72) | 15 | -0.67*** | -0.60*** | -0.60*** | -0.55*** | -0.54*** | 0.04*^NS^* |
|  | 43 | -0.61*** | -0.51*** | -0.42*** | -0.47*** | -0.60*** | -0.15*^NS^* |
| 0-15 cm (*n*=72) | 15 | -0.67*** | -0.55*** | -0.44*** | -0.34** | -0.46*** | -0.17*^NS^* |
|  | 43 | -0.62*** | -0.51*** | -0.27* | -0.26* | -0.57*** | -0.32** |
| 30-45 cm (*n*=72) | 15 | -0.74*** | -0.57*** | 0.39** | 0.07*^NS^* | -0.64*** | -0.59*** |
|  | 43 | -0.70*** | -0.56*** | 0.37** | 0.01*^NS^* | -0.60*** | -0.58*** |

^a^ *^NS^* not significant,* *P*<0.05, ** *P*<0.01, ****P*<0.001
